# The Spectrum of Biopsy‐Proven Renal Diseases in Chinese Elderly Patients and Its Temporal Shift Over 20 Years: A Retrospective Cohort Study

**DOI:** 10.1002/agm2.70093

**Published:** 2026-06-11

**Authors:** Shasha Han, Lengnan Xu, Ying Sun, Fang Fang, Xin Liu

**Affiliations:** ^1^ Department of Nephrology Beijing Hospital, National Center for Gerontology, National Clinical Research Center for Gerontology, The Key Laboratory of Geriatrics of NHC, Institute of Geriatric Medicine, Chinese Academy of Medical Sciences Beijing China; ^2^ Department of Pathology Beijing Hospital, National Center for Gerontology, National Clinical Research Center for Gerontology, The Key Laboratory of Geriatrics of NHC, Institute of Geriatric Medicine, Chinese Academy of Medical Sciences Beijing China

**Keywords:** chronic kidney disease, elderly patients, renal biopsy

## Abstract

**Objective:**

To investigate the longitudinal pathological changes in the spectrum of biopsy‐proven renal diseases among elderly patients over the past 20 years at a single center in China.

**Methods:**

We retrospectively enrolled patients aged ≥ 60 years who underwent renal biopsy at Beijing Hospital between January 2005 and December 2024. Patients were stratified into four 5‐year periods (2005–2009, 2010–2014, 2015–2019, and 2020–2024) based on biopsy date. The frequency and distribution of biopsy‐proven renal diseases across these periods were analyzed.

**Results:**

A total of 594 elderly patients were included, accounting for 29.2% of all native biopsies in our cohort. The mean age was 67.9 ± 5.7 years, with a male‐to‐female ratio of 1.4:1. The proportion of elderly patients undergoing renal biopsy increased significantly across the four periods (19.1%, 22.5%, 33.9%, 38.9%; *χ* ^2^ = 60.160, *p* < 0.01). Nephrotic syndrome (NS) was the most common indication for biopsy, although its proportion declined over time. In contrast, the proportion of biopsies performed for chronic kidney disease (CKD) increased substantially. Primary glomerular nephropathy (PGN), secondary glomerular nephropathy (SGN), and tubulointerstitial nephropathy (TIN) accounted for 54%, 39.9%, and 6.1% of all cases, respectively. The most prevalent cause of PGN was membranous nephropathy (MN), and the leading pathological types of SGN included diabetic nephropathy (DN), antineutrophil cytoplasmic antibody‐associated vasculitis (AAV), and hypertensive nephropathy (HTN). Throughout the study period, the proportion of PGN decreased significantly (64.6%, 64.9%, 54.1%, 42.6%; *χ* ^2^ = 18.5, *p* < 0.001), driven by a decline in MN (34.1%, 39.6%, 32.6%, 23%; *χ* ^2^ = 10.015, *p* = 0.018). Conversely, the proportion of SGN increased markedly (29.3%, 32.4%, 39.4%, and 49.2%; *χ* ^2^ = 13.0, *p* = 0.004), primarily due to rising trends in DN and HTN. Notably, during the period 2020–2024, SGN surpassed PGN as the predominant form of biopsy‐proven renal diseases among elderly individuals.

**Conclusions:**

The utilization of renal biopsy in elderly patients has increased significantly over the past two decades. While NS remains the most common indication, a notable rise in biopsies for CKD has been observed. The pathological spectrum has undergone a significant shift, characterized by a dramatic decrease in PGN (especially MN) and a continuous increase in SGN (predominantly DN and HTN). The predominance shifted to SGN over PGN in biopsy‐proven renal diseases among the elderly in the most recent period.

## Graphical abstract

This 20‐year retrospective study reveals a significant increase in renal biopsy utilization and a pivotal shift in biopsy‐proven renal disease among Chinese elderly patients. Secondary glomerular nephropathy (particularly diabetic nephropathy and hypertensive nephropathy) has surpassed primary glomerular nephropathy as the leading biopsy‐proven diagnoses.

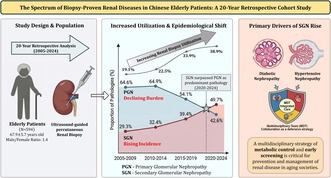

## 1 Introduction

The prevalence of chronic kidney disease (CKD) among elderly individuals has increased significantly, affecting approximately 29% of those aged ≥ 70 years [1]. Age‐related structural and functional alterations in the kidneys, compounded by the presence of comorbidities, heighten the susceptibility to renal diseases in the elderly population. Notably, the spectrum and prevalence of kidney diseases differ substantially between older and younger patients [2, 3]. Renal biopsy remains the gold standard for nephropathy diagnosis, facilitating precise therapeutic strategies and prognostic stratification. When performed under ultrasound guidance with appropriate indications, this procedure has a well‐established safety profile in elderly patients [2, 4, 5, 6]. In recent decades, the growing burden of chronic conditions, such as diabetes and hypertension, has led to dramatic shifts in renal pathological patterns, particularly among elderly individuals. Understanding these evolving trends is critical for developing risk‐stratified interventions and optimizing CKD management. Nevertheless, longitudinal data characterizing temporal changes in renal pathological phenotypes among the Chinese elderly population remain limited. Therefore, we conducted a retrospective cohort study evaluating renal biopsy findings from 594 elderly patients during 2005–2024, to investigate the changing patterns of renal diseases over a 20‐year period.

## 2 Methods

### 2.1 Study Design and Setting

#### 2.1.1 Patients

All native renal biopsies from patients aged ≥ 60 years at Beijing Hospital were obtained between January 2005 and December 2024. Demographic and clinical data were extracted from the hospital's electronic medical record (EMR) system. Patients were stratified into four 5‐year periods, namely, 2005–2009, 2010–2014, 2015–2019, and 2020–2024 on the basis of renal biopsy date. For patients who underwent multiple biopsies, only the first biopsy was included in the analysis. Patients with inadequate biopsies, allograft biopsies, or hereditary nephropathies were excluded.

### 2.2 Clinical Presentation

The primary clinical indications for renal biopsy were as follows: (1) nephrotic syndrome (NS) was defined as proteinuria > 3.5 g/d and serum albumin level < 30 g/L, with or without acute kidney injury (AKI); (2) glomerulonephritis (GN) was defined as the presence of hematuria and/or proteinuria combined with edema and/or hypertension and/or renal dysfunction; (3) AKI was identified by an increase in serum creatinine levels of ≥ 0.3 mg/dL within 48 h, or ≥ 50% from baseline within the preceding 7 days, or a urinary output < 0.5 mL/kg/h for a duration of 6 h; and (4) CKD was defined as an estimated glomerular filtration rate (eGFR) < 60 mL/min per 1.73 m^2^ for more than 3 months and/or detection of bilateral small kidneys or a unilateral small kidney by radiological imaging; (5) asymptomatic urinary abnormality (AUA) was characterized by the presence of hematuria and/or subnephrotic proteinuria without edema, hypertension or renal dysfunction. The biopsy indications were consistent throughout the study period.

### 2.3 Renal Histopathology

All patients underwent ultrasound‐guided percutaneous renal biopsy. All the samples were stained and analyzed via light microscopy (LM) and immunofluorescence staining (for IgG, IgA, IgM, C3, C4, C1q, light chains, and fibrinogen). For LM, paraffin sections were routinely stained with periodic acid‐schiff (PAS), hematoxylin–eosin (HE), and periodic acid‐silver methenamine (PASM). Congo red staining was performed when renal amyloidosis (RA) was suspected. Patients with Hepatitis B Virus (HBV) infection required immunohistochemical examination. Electron microscopy (EM) was performed on selected samples to confirm the LM findings. The histopathological diagnoses were classified according to the revised protocol for histological classification of glomerular diseases issued by the World Health Organization in 1995. The final diagnosis was established through consensus of experienced nephrologists and pathologists, who integrated comprehensive clinical data with histopathological findings.

The renal histopathological diagnoses were categorized into three main groups: primary glomerular nephropathy (PGN), secondary glomerular nephropathy (SGN), and tubulointerstitial nephropathy (TIN). Glomerular diseases combined with tubulointerstitial diseases are categorized as glomerular diseases. PGN includes membranous nephropathy (MN), IgA nephropathy (IgAN), non‐IgA mesangial proliferative glomerulonephritis (MsPGN), minimal change disease (MCD), focal segmental glomerulosclerosis (FSGS), and membranoproliferative glomerulonephritis (MPGN). SGN includes diabetic nephropathy (DN), antineutrophil cytoplasmic antibody (ANCA)‐associated vasculitis (AAV), hypertensive nephropathy (HTN), lupus nephritis (LN), renal amyloidosis (RA), Henoch‐Schönlein purpura nephritis (HSPN), HBV‐associated glomerulonephritis (HBV‐GN), and other renal diseases. The diseases classified as TIN included acute interstitial nephritis (AIN), chronic interstitial nephritis (CIN), and acute tubular necrosis (ATN).

### 2.4 Statistical Analysis

SPSS 27.0 software was used for statistical analysis. Percentages or mean ± SD was used to describe categorical and continuous variables. Comparisons between groups were made via the chi‐square test or Fisher's exact test. Statistical significance was set at a *p* value < 0.05.

## 3 Results

### 3.1 Demographic Characteristics

**FIGURE 1:**
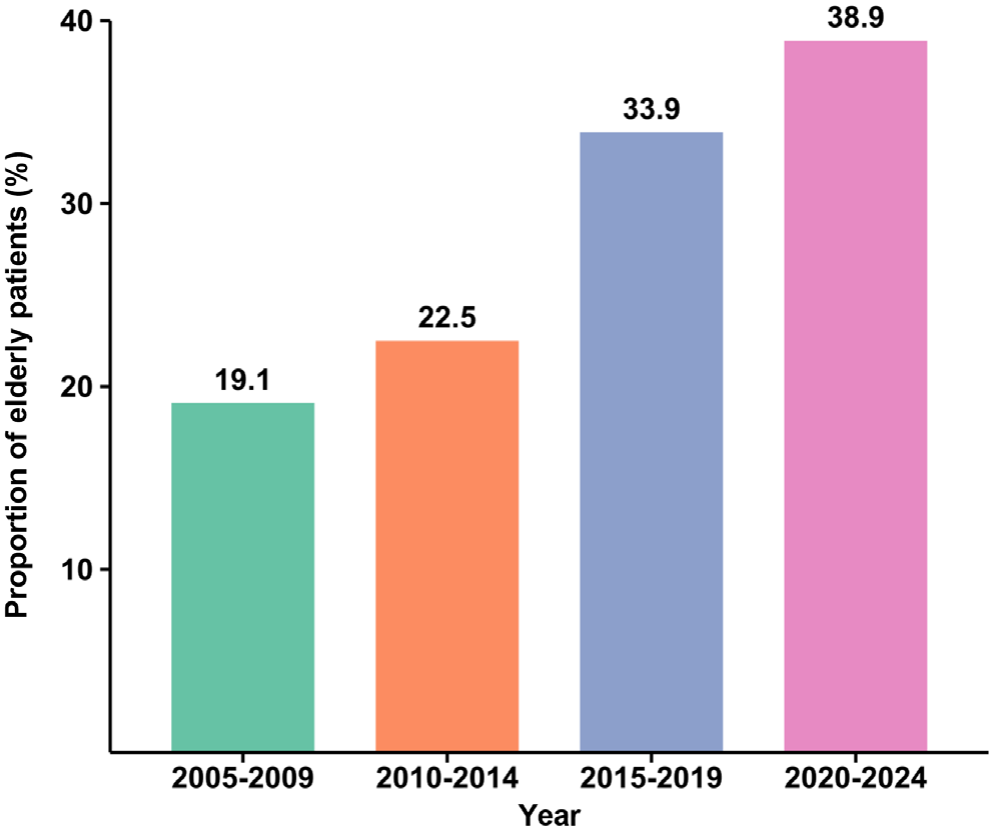
Proportion of elderly patients who underwent renal biopsy across four 5‐year periods (2005–2009, 2010–2014, 2015–2019, 2020–2024).

A total of 2037 native renal biopsies were performed on adults from January 2005 to December 2024, 594 (29.2%) of whom were ≥ 60 years old. The male (*n* = 343) to female (*n* = 251) ratio was 1.4:1.0, with a mean age of 67.9 ± 5.7 years (range: 60–85 years) at the time of renal biopsy among the elderly patients. The proportion of elderly patients who underwent renal biopsy significantly increased across the four 5‐year periods (19.1%, 22.5%, 33.9%, 38.9%; *χ* ^2^ = 60.160, *p* < 0.01) (Figure 1).

### 3.2 Clinical Manifestations

The indications for biopsy are summarized in Table 1. Among elderly patients, NS was the most common indication (268/594, 45.1%), followed by GN (117/594, 19.7%), CKD (100/594, 16.8%), AKI (76/594, 12.8%), and AUA (33/594, 5.6%). In patients biopsied for NS, MN was the most prevalent pathological pattern, representing more than half of all NS cases (141/268, 52.6%), followed by MCD (39/268, 14.6%) and DN (13/268, 4.8%). For patients with GN, IgAN (36/117, 30.8%) and MN (33/117, 28.2%) were the most common pathological findings. The leading causes of CKD were DN (32/100, 32%) and HTN (21/100, 21%). AAV (33/76, 43.4%) was the most common etiology in patients with AKI, followed by TIN (22/76, 28.9%). Among patients with AUA, DN (8/33, 24.2%) and HTN (7/33, 21.2%) were the most common diagnoses.

**TABLE 1:** Histopathological diagnoses associated with primary indications for renal biopsy in elderly patients.

Indications for renal biopsy	*n* (%)	Diagnosis	*n* (%)
Nephrotic syndrome (NS)	268 (45.1)	Membranous nephropathy	141 (52.6)
		Minimal change disease	39 (14.6)
		Diabetic nephropathy	13 (4.8)
		Others	75 (28)
Glomerulonephritis (GN)	117 (19.7)	IgA nephropathy	36 (30.8)
		Membranous nephropathy	33 (28.2)
		ANCA‐associated vasculitis	11 (9.4)
		Others	37 (31.6)
Chronic kidney disease (CKD)	100 (16.8)	Diabetic nephropathy	32 (32)
		Hypertensive nephropathy	21 (21)
		Tubulointerstitial disease	13 (13)
		Others	34 (34)
Acute kidney injury (AKI)	76 (12.8)	ANCA‐associated vasculitis	33 (43.4)
		Tubulointerstitial disease	22 (28.9)
		IgA nephropathy	4 (5.3)
		Others	17 (22.4)
Asymptomatic urinary abnormality (AUA)	33 (5.6)	Diabetic nephropathy	8 (24.2)
		Hypertensive nephropathy	7 (21.2)
		IgA nephropathy	5 (15.2)
		Others	13 (39.4)

**FIGURE 2:**
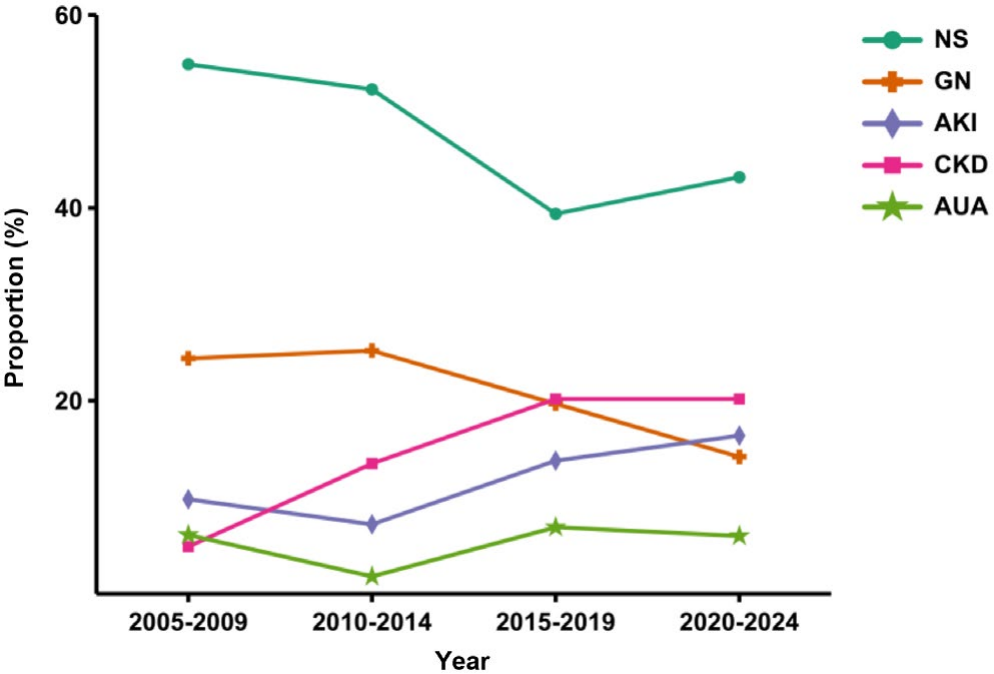
Trends in renal biopsy indications among elderly patients across four 5‐year periods (2005–2009, 2010–2014, 2015–2019, 2020–2024). AKI, acute kidney injury; AUA, asymptomatic urinary abnormality; CKD, chronic kidney disease; GN, glomerulonephritis; NS, nephrotic syndrome.

As shown in Figure 2, across the study periods (2005–2009, 2010–2014, 2015–2019, and 2020–2024), the proportion of NS as an indication for renal biopsy decreased significantly (54.9%, 52.3%, 39.4%, 43.2%; *χ* ^2^ = 8.546, *p* = 0.036). The percentage of GN indications also decreased (24.4%, 25.2%, 19.7%, 14.2%; *χ* ^2^ = 6.773, *p* = 0.079). Conversely, the proportion of CKD indications increased substantially (4.9%, 13.5%, 20.2%, 20.2%; *χ* ^2^ = 12.490, *p* = 0.006). During the periods of 2015–2019 and 2020–2024, CKD surpassed GN as the second most common indication for renal biopsy, with proportions of 20.2% versus 19.7% and 20.2% versus 14.2%, respectively. The percentage of AKI indications increased (9.8%, 7.2%, 13.8%, 16.4%; *χ* ^2^ = 6.091, *p* = 0.107), while the trend did not reach statistical significance. The proportion of AUA remained relatively stable (6.1%, 1.8%, 6.9%, and 6.0%; *χ* ^2^ = 3.829, *p* = 0.240).

### 3.3 Distribution of Pathological Patterns

**FIGURE 3:**
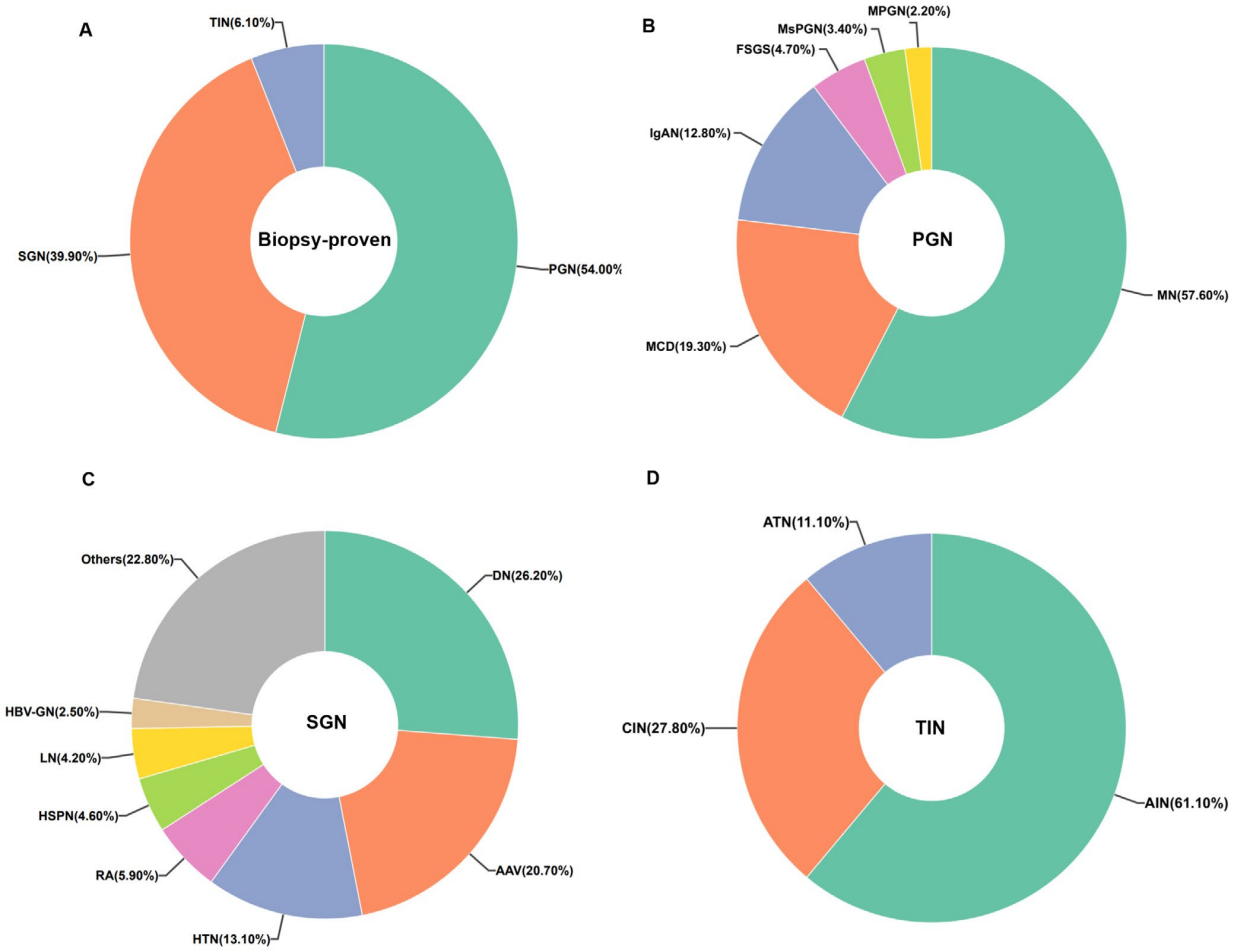
Distribution of biopsy‐proven renal diseases in elderly individuals (A), and frequencies of pathological diagnoses within categories: Primary glomerular nephropathy (PGN) (B), secondary glomerular nephropathy (SGN) (C), and tubulointerstitial nephropathy (TIN) (D). AAV, antineutrophil cytoplasmic antibody‐associated vasculitis; AIN, acute interstitial nephritis; ATN, acute tubular necrosis; CIN, chronic interstitial nephritis; DN, diabetic nephropathy; FSGS, focal segmental glomerulosclerosis; HBV‐GN, Hepatitis B virus‐associated glomerulonephritis; HSPN, Henoch‐Schönlein purpura nephritis; HTN, hypertensive nephropathy; IgAN, IgA nephropathy; LN, lupus nephritis; MCD, minimal change disease; MN, membranous nephropathy; MPGN, Membranoproliferative glomerulonephritis; MsPGN, mesangial proliferative glomerulonephritis; RA, renal amyloidosis.

The distribution of pathological patterns was summarized in Figure 3. PGN was the most prevalent category, accounting for 54% (321/594) of the elderly patients, followed by SGN (237/594, 39.9%) and TIN (36/594, 6.1%) (Figure 3A). Among patients with PGN, MN constituted the most common type (185/321, 57.6%), followed by IgAN (62/321, 19.3%), MCD (41/321, 12.8%), FSGS (15/321, 4.7%), MsPGN (11/321, 3.4%), and MPGN (7/321, 2.2%) (Figure 3B). Among the patients with SGN, DN was the leading cause, accounting for 26.2% (62/237), followed by AAV (49/237, 20.7%) and HTN (31/237, 13.1%). The frequencies of RA, HSPN, LN and HBV‐GN were 5.9% (14/237), 4.6% (11/237), 4.2% (10/237), and 2.5% (6/237), respectively (Figure 3C). Among the elderly patients diagnosed with TIN, 61.1% (22/36) had AIN, 27.8% (10/36) had CIN, and 11.1% (4/36) had ATN (Figure 3D).

**FIGURE 4:**
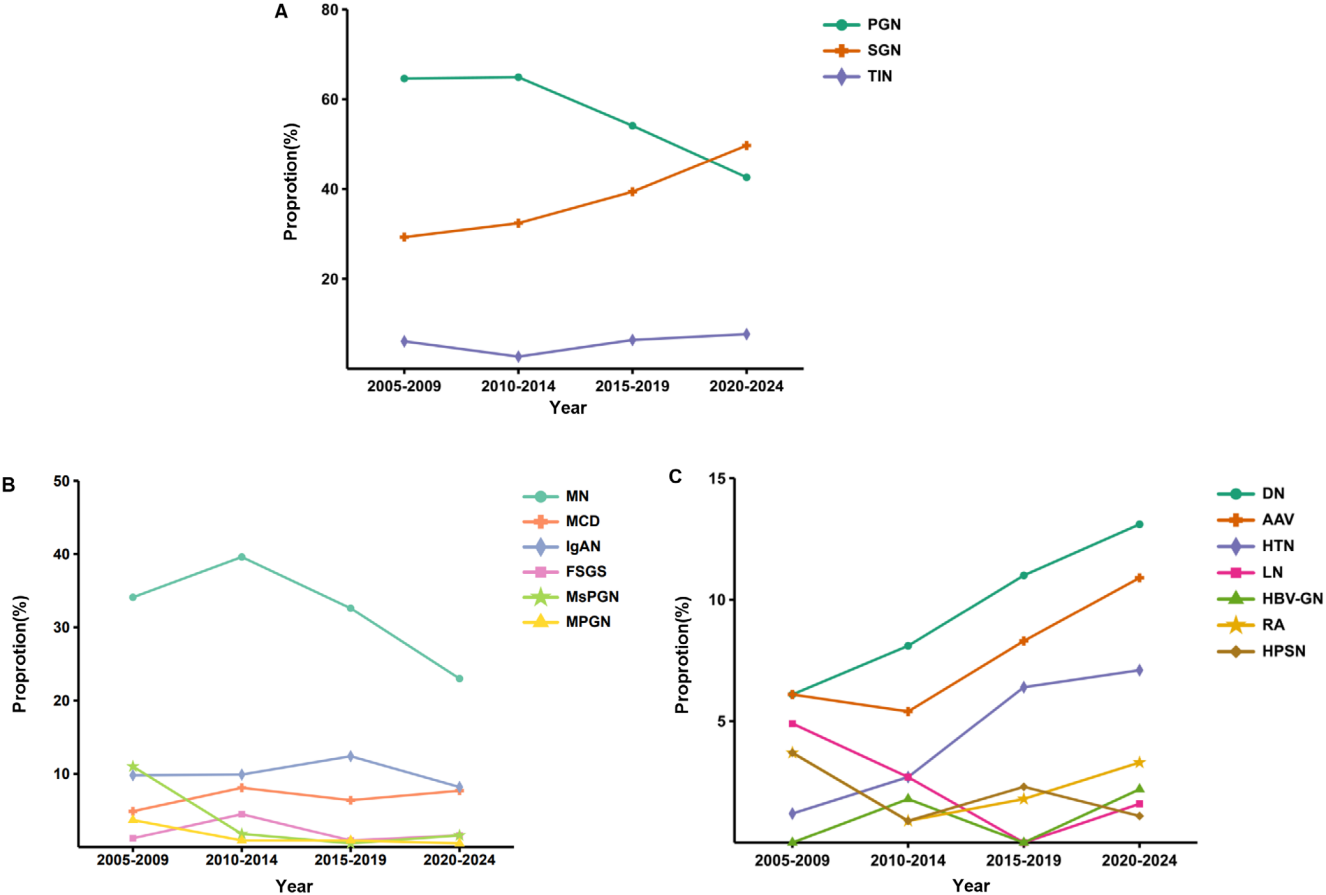
Trends in biopsy‐proven renal diseases in elderly patients across the four 5‐year periods (A) and trends within primary glomerular nephropathy (B) and secondary glomerular nephropathy (C). AAV, antineutrophil cytoplasmic antibody‐associated vasculitis; DN, diabetic nephropathy; FSGS, focal segmental glomerulosclerosis; HBV‐GN, Hepatitis B virus‐associated glomerulonephritis; HSPN, Henoch‐Schönlein purpura nephritis; HTN, hypertensive nephropathy; IgAN, IgA nephropathy; LN, lupus nephritis; MCD, minimal change disease; MN, membranous nephropathy; MPGN, membranoproliferative glomerulonephritis; MsPGN, mesangial proliferative glomerulonephritis; PGN, primary glomerular nephropathy; RA, renal amyloidosis; SGN, secondary glomerular nephropathy; TIN, tubulointerstitial nephropathy.

The proportion of PGN decreased significantly across the four periods of 2005–2009, 2010–2014, 2015–2019, and 2020–2024 in elderly patients (64.6%, 64.9%, 54.1%, 42.6%; *χ* ^2^ = 18.5, *p* < 0.001) (Figure 4A). This decline was driven primarily by a marked reduction in MN (34.1%, 39.6%, 32.6%, 23%; *χ* ^2^ = 10.015, *p =* 0.018) (Figure 4B). Conversely, the proportion of SGN increased significantly (29.3%, 32.4%, 39.4%, and 49.2%; *χ* ^2^ = 13.0, *p* = 0.004) (Figure 4A). Among patients with biopsy‐proven SGN, the proportions of DN and HTN tended to increase, although these differences did not reach statistical significance (6.1%, 8.1%, 11%, and 13.1%; *χ* ^2^ = 3.776, *p* = 0.286; 1.2%, 2.7%, 6.4%, and 7.1%; *χ* ^2^ = 5.958, *p* = 0.106) (Figure 4C). No significant change was observed in the proportion of patients with TIN (6.1%, 2.7%, 6.4%, and 8.2%; *χ* ^2^ = 3.592, *p* = 0.309) (Figure 4A).

## 4 Discussion

In this retrospective analysis, we examined the changing spectrum of biopsy‐proven renal diseases among 594 elderly patients over the past 20 years. Elderly patients comprised 29.2% of all biopsies at our center, which is much higher than the reported range of 6.3% to 17.1% in previous Chinese studies [2, 7, 8, 9, 10]. This discrepancy likely reflects the status of our hospital as a national geriatric medical center, where patients aged ≥ 60 years comprised more than 50% of hospitalizations during the study period. We observed a doubling increase in the proportion of elderly patients who underwent renal biopsy, increasing from 19.1% in 2005–2009 to 38.9% in 2020–2024. This trend may be attributed to several factors. Firstly, with established safety profiles and low complication rates [2, 4, 5], renal biopsy under ultrasound guidance has become a standard and valuable diagnostic procedure routinely performed in elderly patients. Secondly, accelerated population aging in China, with the proportion of citizens aged ≥ 60 years increasing from 11.3% in 2005 to 21.1% in 2023, has contributed to a marked increase in CKD prevalence among elderly individuals.

NS was the most common biopsy indication in our elderly patients, followed by GN and CKD, which is consistent with the findings of previous studies [2]. Notably, the proportion of biopsies performed for NS progressively declined, whereas biopsies for CKD increased substantially, surpassing GN as the second most frequent indication starting in 2015. This shift may be explained by the increasing prevalence of DN and HTN as the predominant etiologies for CKD biopsies in our cohort, driven by socioeconomic improvements. Additionally, nationwide CKD screening programs may have enhanced the recognition of early‐stage disease, expanding biopsy eligibility for early CKD [11].

Our findings revealed that PGN accounted for 54% of all biopsy samples. A gradual reduction has been observed in the proportion of PGN, despite its continued status as the most prevalent cause of biopsy‐proven renal diseases among elderly patients in the periods of 2005–2009 (64.6%), 2010–2014 (64.9%), and 2015–2019 (54.1%). MN was the most common PGN, which is similar to the findings of previous reports [3, 7, 8, 12]. Some studies have shown that MN tends to increase [13, 14]. In our study, a similar increasing trend of MN was observed in 2005–2009 (34.1% of PGN) and 2010–2014 (peak at 39.6% of PGN), whereas the proportion of MN subsequently began to decline significantly (32.6% in 2015–2019 and 23% in 2020–2024). This decline may be attributed to two main factors. First, environmental interventions: air pollution can promote MN development through oxidative stress and extracellular vesicle‐mediated signaling [15]. MN has a relatively high incidence rate in heavily polluted northern cities. Xu et al. demonstrated that each 10 μg/m^3^ increase in the PM2.5 concentration was associated with 14% greater odds of MN [16]. In Beijing, the PM2.5 concentration has significantly decreased from 89.5 μg/m^3^ in 2013 to 30 μg/m^3^ in 2022 [17], potentially reducing the incidence of environmentally related MN. Second, serological diagnosis: the growing popularity of anti‐M‐type phospholipase A2 receptor (PLA2R) antibody testing has allowed for the direct clinical diagnosis of MN in some high‐risk elderly patients, who may have been exempted from renal biopsy, leading to selection bias in biopsy cases. It is important to note that these explanations are based on ecological correlations and plausible mechanisms; however, the single‐center design of our study limits our ability to draw definitive causal inferences.

SGN represented 39.9% of the total cases, with its proportion increasing from 29.3% in 2005–2009 to 49.2% in 2020–2024. Notably, during the most recent period (2020–2024), SGN surpassed PGN as the predominant cause of biopsy‐proven renal disease in elderly individuals. The most prevalent SGN types were DN, AAV, and HTN. DN and HTN exhibited persistent increases during the study period. The kidneys are major target organs affected by diabetes and hypertension, with 30%–40% of patients with diabetes developing DN [18] and approximately 20% of patients with hypertension progressing to HTN [19]. The expanding burden of DN and HTN has been observed globally, closely correlating with the increasing prevalence of diabetes (9.7% in 2007 to 12.8% in 2021 [20]) and hypertension (18.8% in 2002 to 23.2% in 2019 [21]) in China over recent decades. Zhang et al. first reported that diabetes‐associated CKD surpassed glomerulonephritis‐associated CKD on the basis of a national hospitalized patient database in China [22]. These trends indicate that metabolic disorder‐related renal diseases, particularly DN, are projected to gain increasing prominence within the SGN spectrum. AAV constituted the second most prevalent cause of SGN, which is consistent with other Chinese reports [2]. Some studies have indicated that AAV is the most common SGN in elderly individuals [4]. Moreover, AAV has emerged as the predominant etiology of AKI in elderly patients. Given the prominent status of AAV among elderly patients, ANCA serologic screening should be implemented in clinical practice, particularly in those presenting with AKI.

This study had several limitations. Firstly, the biopsy‐dependent diagnostic framework may not be applicable to the general population and may underestimate the true burden of SGN, as some very elderly patients or those with mild clinical manifestations or comorbid malignancies are unwilling to undergo biopsies. Secondly, as a single‐center retrospective analysis, the lack of longitudinal follow‐up data limits our ability to assess long‐term outcomes, disease progression patterns, and prognostic factors. Multicenter prospective studies with follow‐up data are needed to elucidate the roles of various factors in the frequency of kidney diseases.

## 5 Conclusion

Our study demonstrated a significant increase in renal biopsy utilization among elderly patients over the past 20 years. A substantial transformation in the pathological spectrum has occurred, characterized by a declining burden of PGN (predominantly due to reduced MN) alongside a rising incidence of SGN (driven by increases in DN and HTN). Notably, over the past five years, SGN has exceeded PGN as the leading cause of biopsy‐proven kidney disease in elderly individuals. This epidemiological shift reflects the complex interplay among population aging, escalating metabolic disorders, and socio‐environmental influences on renal pathogenesis. Consequently, renal biopsy retains critical diagnostic value in elderly individuals, particularly in those with complex multimorbidity. To address these challenges in an aging society, establishing multidisciplinary collaborative frameworks and optimizing management through rigorous metabolic control and population‐based early detection programs will be pivotal in mitigating the progression of renal disease.

## Author Contributions

Conception and design: Xin Liu; histopathological analysis and specimen validation: Fang Fang; literature search and data acquisition: Lengnan Xu, Ying Sun, and Shasha Han; data analysis and statistical analysis: Lengnan Xu and Shasha Han; manuscript preparation: Shasha Han; manuscript editing and manuscript review: Xin Liu. All authors read and approved the final manuscript.

## Funding

This study was supported by National High Level Hospital Clinical Research Funding under Grant No. FFBJ‐2024‐253, the National Natural Science Foundation of China under Grant No. 82400820, Beijing Hospital Clinical Research 121 Project under Grant BJ‐2019‐197, and State Key Laboratory of Respiratory Health and Multimorbidity, State Key Laboratory Special Fund 2060204.

## Ethics Statement

This was a retrospective study using clinical and pathological data, and it did not involve further invasive intervention, treatment, or costs to patients. All patients' records were deidentified and analyzed anonymously. The study was conducted in compliance with the Declaration of Helsinki (2013) and was approved by the ethics committee of Beijing Hospital (Approval No. 2022BJYYEC‐209‐02). The study was conducted in accordance with local legislation and institutional requirements.

## Consent

Written informed consent was not required in accordance with national legislation and institutional guidelines.

## Conflicts of Interest

The authors declare no conflicts of interest.

## Data Availability

The data that support the findings of this study are available on request from the corresponding author. The data are not publicly available due to privacy or ethical restrictions.
